# Molecular phylogenetic analysis of bacterial community and characterization of Cr(VI) reducers from the sediments of Tantloi hot spring, India

**DOI:** 10.1186/2046-9063-10-7

**Published:** 2014-09-02

**Authors:** Preeti Jain, Hasan Mahmud Reza, Subrata Pal

**Affiliations:** 1 Department of Pharmaceutical Sciences, North South University, Dhaka, Bangladesh; 2 Department of Life Science and Biotechnology, Jadavpur University, Kolkata, India

**Keywords:** Phylogenetic analysis, Bacterial community, Hot spring, Chromium reduction, Bioremediation

## Abstract

**Background:**

A geothermal ecosystem located at Tantloi, India has been found to be an interesting habitat for microbes of diverse nature. However, the microbial diversity of this habitat is poorly explored. In this study, a detailed phylogenetic study has been carried out to understand the bacterial diversity of this habitat and to identify prospective metal reducers using culture independent approach. The bacterial diversity of the sediments, which contain undetectable levels of Cr(VI), was analysed with respect to chromium reduction and the strains highly resistant to and efficiently reducing chromium under aerobic conditions were isolated and characterized.

**Results:**

16S rRNA gene sequence analysis of Tantloi hot spring microbial community revealed a significant bacterial diversity represented by at least ten taxonomic divisions of *Bacteria* with clear predominance of *Thermus*. Similar sequence analysis of rRNA gene library clones derived from bacterial consortia enriched from sediments in presence of Cr(VI) revealed the abundance of the family *Bacillaceae*. Under aerobic conditions at 65°C, the consortia reduced 1 mM of Cr(VI) completely within 24 h and 5 mM in 6 days. A complete reduction of 1 mM Cr(VI) has been shown by five of our isolates within 36 h. 16S rRNA gene sequences of all the isolates showed high degree of similarity (97-99%) to *Bacillaceae* with ten of them being affiliated to *Anoxybacillus*. Crude extract as well as the soluble fraction from isolates TSB-1 and TSB-9 readily reduced Cr(VI); TSB-1 showed higher chromium reductase activity.

**Conclusion:**

Most of the Tantloi Spring Bacterial (TSB) sequences analyzed in different taxonomic divisions could be related to representatives with known metabolic traits which indicated presence of organisms involved in redox processes of a variety of elements including iron, sulphur and chromium. Approximately 80% of the sequences obtained in this study represented novel phylotypes indicating the possibility of discovery of bacteria with biotechnologically important new biomolecules. Again, highly chromium-resistant and remarkably active Cr(VI)-reducing *Anoxybacillus* strains isolated in this study could serve as potential candidates for designing chromium bioremediation strategies at high temperatures and also at high chromium concentrations.

## Background

Molecular, physiological and ecological studies of thermal biosystems can provide insight into many of the fundamental processes and questions in biology [1,2]. Several studies have revealed that microbial diversity retrievable by standard cultivation techniques covers only a minor fraction, usually less than 1%, of the diversity present in complex environmental samples such as soil [3]. In fact, most naturally occurring microbes exist in complex communities, and have never been cultivated or characterized in the laboratory [4]. Investigations on microbial communities of tropical environments have shown that tropics are richer in microbial diversity than boreal or temperate environments and contain remarkable bacterial diversity [5-7]. It is quite known that India is rich in its diversity, and this undoubtedly includes microbial diversity. However, only sporadic attempts have been made to isolate thermophiles and understand ecological roles, diversity and biotechnological applications of microorganisms from thermal ecosystems of India using culture independent approaches [8,9]. Whereas cultivation-dependent studies are valuable for isolating novel organisms and exploring their properties; the cultivation independent methods offer a more comprehensive assessment of microbial diversity [4,10,11]. Both the sediment and the soil probably represent some of the most complex microbial habitats on the earth. There may be several thousand species of bacteria in 1 g of soil [3,12] which can be studied using various approaches to understand the genetic diversity of microbial populations of different microbial habitats [13].

Chromium-reducing microorganisms have attracted special attention due to their potential for bioremediation. Soluble Cr(VI) is extremely toxic and exhibits mutagenic and carcinogenic effects on biological systems due to its strong oxidizing nature [14]. Reduction of the hexavalent form produces water-insoluble and less mobile Cr(III), which is less toxic due to a decrease in bioavailability [15,16]. The widespread use of chromium oxyanions in industries such as leather tanning, metallurgy, electroplating, petroleum refining, textile manufacturing and pulp production has resulted in large quantities of chromium being discharged into the environment. The discharge of heavy metals into the environment by different industries constitutes one of the major causes of land and water pollution [17].

Tantloi in the Jharkhand state of India harbors a geothermal ecosystem having microbes of diverse nature. Water flowing in shallow streams reaches a temperature as high as 70°C. Layers of reddish precipitate, dominated by Fe^3+^, are seen to be forming on the sides. Beneath the reddish layer has been found darker soil containing iron in the reduced form. In this study, we aimed to explore the bacterial community present in Tantloi hot spring to gain knowledge about nature and possible roles of microorganisms present therein with respect to redox phenomena. Considering that chromium-reducing bacteria had a prospect in bioremediation applications, enrichment consortia were derived from the native samples and their chromium-reducing properties were studied to isolate highly resistant strains that can efficiently reduce toxic chromium (VI). Furthermore, cell-free extracts of the most active isolate were used to study hexavalent chromium reduction in vitro.

## Results

### Hot spring sediments

The sediment temperature varied between 68-69°C and the pH of water at corresponding points between 8.0-9.0. The total chromium content was 300 mg kg^-1^ (dry weight) while, free soluble chromium level and Cr(VI) content were below detection level.

### 16S rDNA library

The yield of DNA obtained from sediments was about 5 μg/g of sample (wet weight). Although DNA isolated from sediment samples initially was not amplifiable and contained a brownish tinge possibly due to coextraction of humic contaminants, two rounds of purification through QIAGEN Genomic tip resulted in almost colorless DNA solution. PCR amplification was standardized with respect to template concentration. Addition of more than 1 ng of template DNA in 50 μl of PCR reaction mix resulted in complete inhibition of the reaction. Template concentration of 600 pg per reaction gave the best yield.

**Figure 1:**
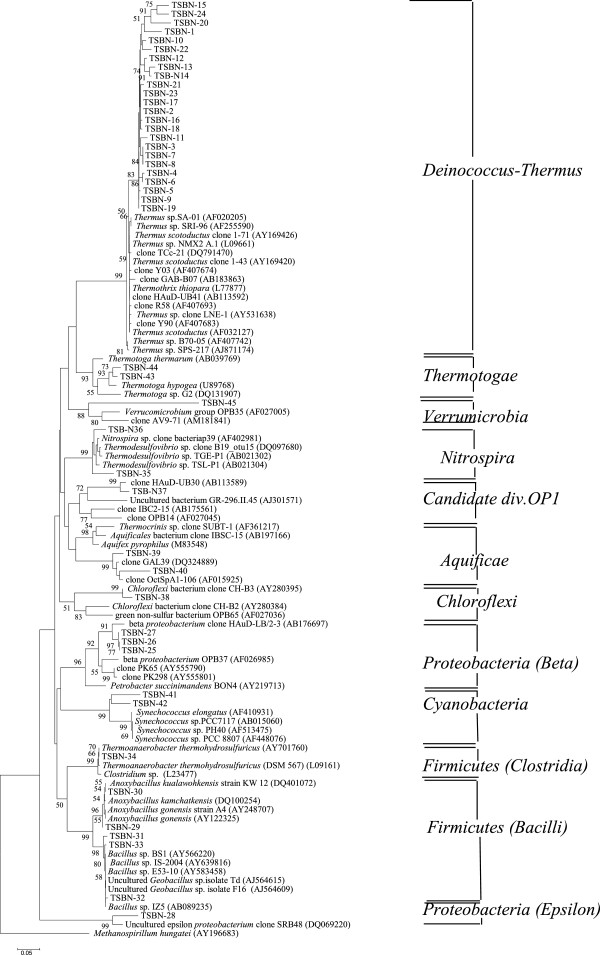
**Phylogenetic tree deduced from the 16S rRNA gene sequences of Tantloi hot spring sediments by neighbor-joining algorithm.** Reference sequences were chosen to represent the broadest diversity of *Bacteria*. The archeon *Methanospirillum hungatei* was used as outgroup. Branch points supported with bootstrap values ≥ 50% are indicated. The scale below shows substitution per site.

A 16S rRNA gene library was generated using the 1.3 kb gel purified amplification product from native soil samples. Out of 70 such clones sequenced, 45 were found to be unique. BLAST analysis revealed that all the sequences were bacterial in nature. They could be classified into ten taxonomic divisions (Figure 1). Although the majority of the clones (30 out of 70) belonged to the division *Deinococcus-Thermus*, there were representatives of the divisions *Proteobacteria, Firmicutes, Nitrospira, Chloroflexi, Aquificae, Cyanobacteria, Thermotogae and Verrumicrobia*. When the phylotypes were compared with the closest organisms of known metabolic traits within the respective groups, a remarkable metabolic diversity was apparent in the native bacterial community (Table 1). The efficiency of lysis of soil bacteria during DNA extraction could be relied upon since a sizeable number of *Bacillus* species that are relatively resistant to lysis could be identified [18]. A neighbor-joining tree constructed using an archeon *Methanospirillum* as outgroup with MEGA 3.1 software is shown in Figure 1.

**Table 1:** Predicted energy metabolism of some native phylotypes

**Clone no.**	**Closest group**	**Representatives with known energy metabolism closest to native clones**	**Predicted energy metabolism**
TSBN-1 to TSBN-21	*Thermus-Deinococcus* group	*Thermus* SA-01 (AF020205)	Reduction of Fe, S, Mn, Cr, U and Co under anaerobic condition
		*Thermus* sp. NMX2 A.1 (L09661)	Dissimilatory reduction of Fe, Mn, Cr,U and Co under anaerobic condition, sulfur oxidation under aerobic conditions
TSBN-22 to TSBN-24	β-*Proteobacteria*	*Thermothrix thiopara*	Sulfur oxidation
TSBN-28	ϵ-*Proteobacteria*	Clone IRR-DS7-17. \	Nitrate, sulfate and ferric iron reduction
TSBN-34	*Clostridia*	*Thermoanaerobacter siderophilus* (AF120479)	Anaerobic Fe(III)-reduction
		*Thermoanaerobacter ethanolicus* strain X513 (AF542520)	Dissimilatory reduction of Fe, Mn, Cr,U and Co under anaerobic condition
TSBN-35, TSBN-36	*Nitrospira*	*Thermodesulfovibrio* sp. TGE-P1 (AB021302)	Sulfate reduction
		*Thermodesulfovibrio yellowstonii* (AB231858)	Sulfate reduction
TSBN-39	*Aquificales*	*Thermocrinis* sp. H7L1B (AJ320222)	Utilization of sulfur

### Enrichment culture

**Figure 2:**
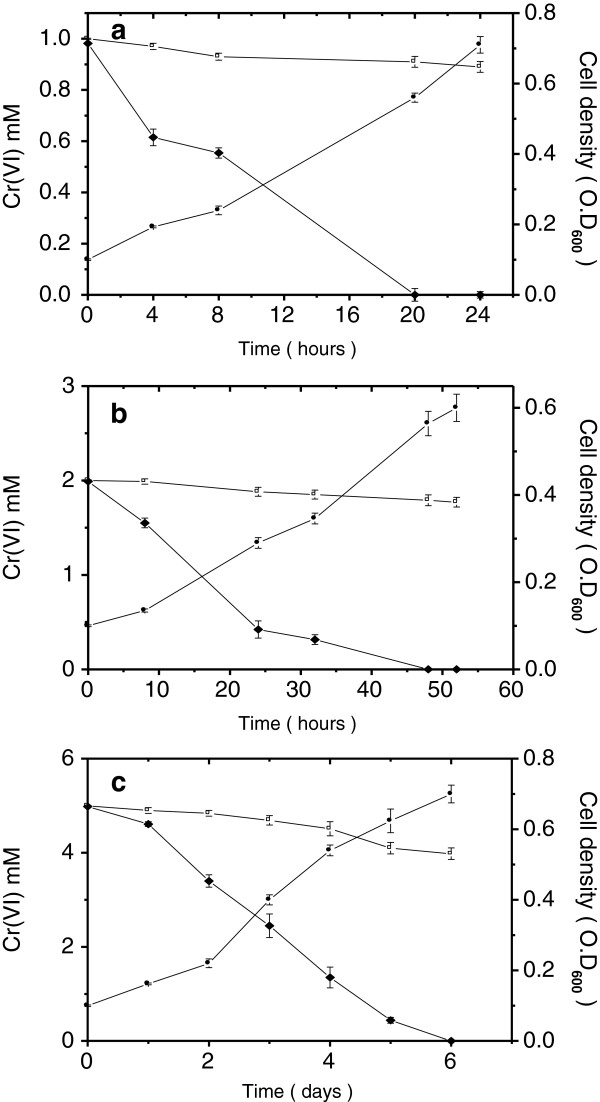
**Aerobic growth and Cr(VI) reduction by enrichment consortium for 1 mM (a) 2 mM (b) and 5 mM (c) initial concentrations of Cr(VI).** In each frame (•) indicates growth of cells as measured by OD_600 nm_, (□) reduction by medium alone and (♦) reduction by consortium. Error bars indicate standard deviation of triplicate experiments.

Sequence analysis of the 16S rRNA gene clones revealed a predominance of *Thermus* in the native sample. Interestingly, two of the close relatives were *Thermus* SA-01 and *Thermus* sp. NMX2 A.1 which were earlier reported to be capable of reducing a number of metals including chromium [19]. We analyzed the chromium reduction ability of the consortia enriched in presence of Cr(VI) and observed that under anaerobic conditions the consortia grew well at 0.3 mM initial Cr(VI) concentration. At 0.5 mM concentration, growth was slower whereas at 1 mM initial Cr(VI) concentration, no growth was observed even after 10 days of incubation. Apparently, 1 mM Cr(VI) was toxic to the bacterial population. Besides, anaerobic enrichment consortia reduced 0.3 mM Cr(VI) completely within 3 days while at 0.5 mM initial concentration, complete reduction occurred only after 15 days (data not shown).Consortia incubated under aerobic conditions, in contrast, showed bacterial growth and chromium reduction at different Cr(VI) concentrations up to 5 mM (Figure 2). At 1 mM of initial Cr(VI) concentration, the reduction was complete within 24 h with no inhibitory effect on bacterial growth and 2 mM Cr(VI) was completely reduced in 48 h although the bacterial growth was slower. At 5 mM initial Cr(VI) concentration, complete reduction required about 6 days and bacterial growth was conspicuously retarded. Even at 20 mM concentration, reduction was observed, although much slowly, 75% being reduced in 25 days (data not shown). It may be noted that there was negligible Cr(VI) reduction in uninoculated medium or medium inoculated with autoclaved soil.

**Figure 3:**
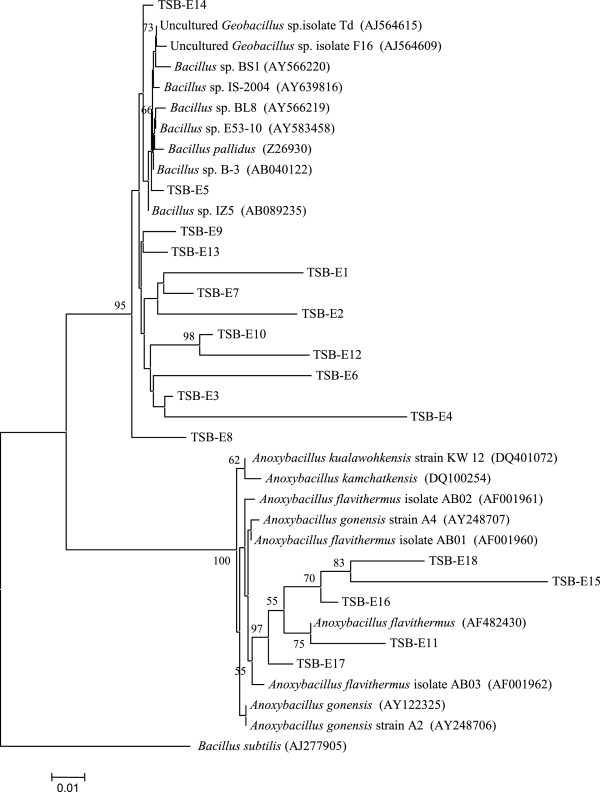
**Phylogenetic tree deduced from the 16S rRNA gene sequences as obtained from the enrichment consortium by neighbor joining algorithm.***Bacillus subtilis* was used as outgroup. Branch points supported with bootstrap values ≥ 50% are indicated. The scale below shows substitution per site.

We randomly picked 30 16S rRNA gene clones from the library derived from the enrichment consortia and sequenced. Of them 18 were found to be unique. Carrying out BLAST analysis and constructing phylogenetic tree 14 of the unique sequences could be affiliated to *Bacillus* while 4 to *Anoxybacillus* (Figure 3). The tree topology was virtually similar for different algorithms as mentioned in Methods.

### Cr(VI) reduction by pure isolates

11 Pure isolates were obtained from the consortia on LB-agar medium containing 5 mM Cr(VI) and 10 of them were analyzed for chromium-resistance and chromium-reducing properties. All the isolates grew on LB-agar medium containing up to 5 mM Cr(VI) when incubated at 65°C. Visible colonies were observed on agar medium containing 10 mM Cr(VI) in 48 h with all the isolates except three, namely, TSB-4, TSB-5 and TSB-10, which formed such colonies only after 3 days of incubation. On plates containing 20 mM Cr(VI), colonies were formed by TSB-1, 2, 3, 7, 8, 9 and 11 after 7 days of incubation at 65°C while isolates, TSB-1, 2 and 9 formed colonies on 30 mM Cr(VI) after 14 days of incubation. To be noted that we analyzed the strain TSB-6 in our previous study [20].

**Figure 4:**
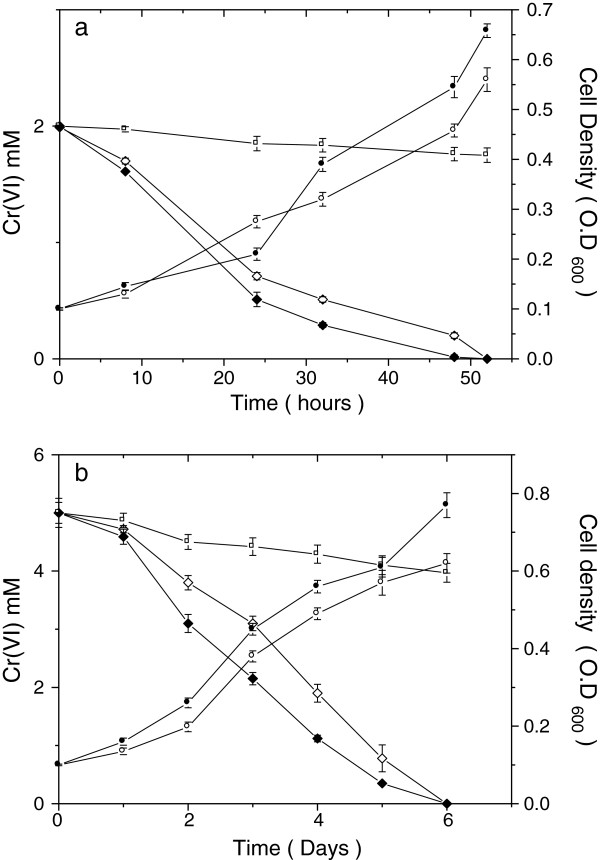
**Growth pattern and Cr(VI) reduction by isolates TSB-1 and TSB-9 for initial Cr(VI) concentrations of (a) 2 mM and (b) 5 mM.** In each frame growth as measured by OD_600 nm_ for TSB-1 (•) and TSB-9 (ο) and chromium reduction by TSB-1 (♦), TSB-9 (◇) and medium alone (□) have been shown. Error bars indicate standard deviation of triplicate experiments.

Cr(VI) reduction activity of the isolates under aerobic conditions is shown in Table 2. TSB-1, 2, 5, 9 and 11 reduced 1 mM Cr(VI) completely within 36 h, four other did so in 48 h while TSB-8 did not reduce Cr(VI) completely even after 3 days of incubation. TSB-1 and 9 showed the high reduction activity amongst the isolates that completely reduced Cr(VI) (Table 2) and their reduction potential was studied at higher initial concentrations. Both TSB-1 and TSB-9 reduced 2 mM Cr(VI) completely within 52 h at about the same rate while 5 mM Cr(VI) was reduced completely in 6 days by both the isolates, however, better growth and reduction potential was found with TSB-1 (Figure 4).

**Table 2:** **Growth of and Cr(VI) reduction by chromium-resistant isolates at starting cell density O.D.**_**600 **_**ca.0.1 after 36 hours incubation**

**Isolate no.**	**A**_**600**_	**Residual Cr(VI)**^**a **^**(mM)**	**% Cr(VI) reduced**	**Cr(VI) transformed per A**_**600 **_**of growth**
TSB-1	0.56	0	100	1.79
TSB-2	0.65	0	100	1.54
TSB-3	0.3	0.4	60	2.00
TSB-4	0.251	0.6	40	1.59
TSB-5	0.690	0	100	1.45
TSB-7	0.325	0.65	35	1.08
TSB-8	0.202	0.68	32	1.58
TSB-9	0.0.596	0	100	1.68
TSB-10	0.42	0.66	34	0.81
TSB-11	0.695	0	100	1.44

**Figure 5:**
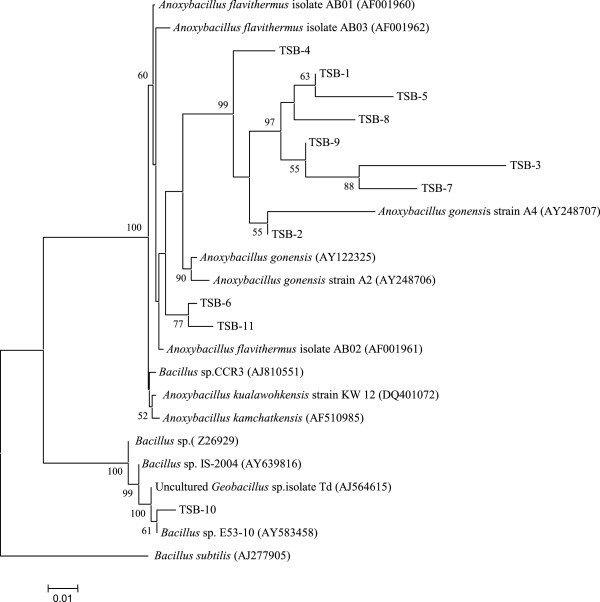
**Phylogenetic tree of chromium-resistant pure isolates by neighbor joining algorithm.***Bacillus subtilis* was used as outgroup. Branch points supported with bootstrap values ≥ 50% are indicated. The scale below shows substitution per site.

BLAST analysis was carried out with 16S rRNA gene sequences of all the 11 isolates. All of them had high levels (97-99%) of sequence similarity with microorganisms of the family *Bacillaceae*; in fact, ten out of the eleven could be affiliated to the genus *Anoxybacillus* while TSB-10 had sequence similarity (97%) with *Bacillus* IS-2004 and uncultured *Geobacillus* isolate Td. Phylogenetic tree constructed with 16S rRNA gene sequences using neighbor joining, parsimony and minimum evolution algorithms showed that the isolates clustered closely with the members of the genus *Anoxybacillus* except for TSB-10 which clustered with members of the genus *Bacillus*/*Geobacillus*. A neighbor-joining tree with *Bacillus subtilis* as outgroup is shown in Figure 5.

### Localization of chromate reductase activity

**Figure 6:**
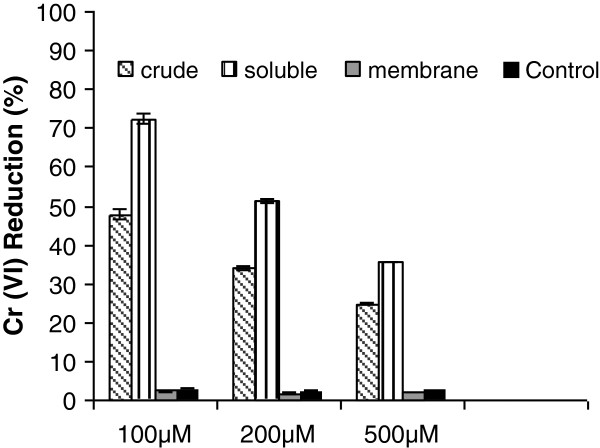
**Percentage Cr(VI) reduction by cell extracts of TSB-1 with NADH as electron donor.** Percentage reduction is shown at different initial concentrations of Cr(VI) as observed after 90 minutes of incubation. Error bars indicate standard deviation of triplicate experiments.

Chromium-reduction activity of TSB-1 was further characterized with respect to its cell-free extract. Crude extracts were fractionated by ultracentrifugation into soluble and membrane components. For in vitro assay of Cr(VI) reductase activity, NADH was used as the electron donor. The crude extract as well as the soluble fraction readily reduced Cr(VI). When equal amounts of protein were used in the reactions, the soluble fraction showed higher activity at all the tested initial Cr(VI) concentrations (Figure 6). This might be due to some inhibitor present in the crude extract. Reduction by the membrane fraction was found to be negligible. Similar experiments with TSB-2 and TSB-4 also indicated that Cr(VI) reduction activity was associated with the soluble fraction (data not shown).

**Figure 7:**
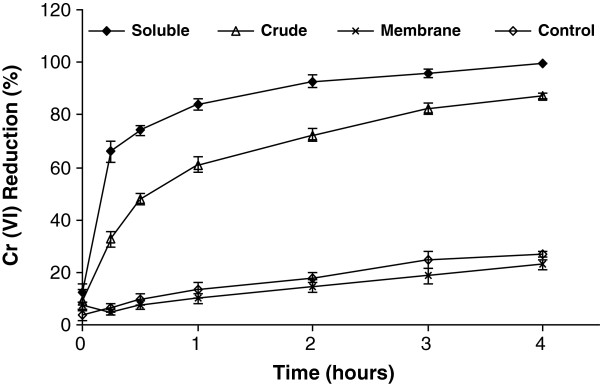
**Time course of Cr(VI) reduction by cell extracts of TSB-1 with NADH as electron donor.** The concentration of Cr(VI) used in this study was 50 μM. Error bars indicate standard deviation of triplicate experiments.

The time course of Cr(VI) reduction by different fractions of the cell-free extract was carried out with NADH as the electron donor taking the initial Cr(VI) concentration of 50 μM (Figure 7). The specific activity calculated on the basis of initial 15 min reaction was 6.88 U mg^-1^ protein for the crude extract and 15.67 U mg^-1^ protein for the soluble fraction (1 unit is defined as the amount of enzyme that converts 1 nmol of Cr(VI) min^-1^ at experimental conditions as described in Methods).

## Discussion

The current study has revealed a significant metabolic diversity represented by at least ten taxonomic divisions of *Bacteria* with clear predominance of *Thermus* in Tantloi hot spring. Approximately 80% of the sequences obtained in this study represented novel phylotypes that had less than 97% similarity with known sequences. If we have to understand the roles of hitherto uncultivated microbes in an ecosystem, it will be necessary to infer physiological properties of organisms based on molecular data. An rRNA sequence in the absence of additional information provides little insight into the metabolism of the organisms that contributed the sequences. If, however, phylogenetic analysis groups the sequence with those of organisms that all possess a particular trait, then that trait is likely to occur in the organism which is otherwise known only by a sequence [21]. Hence, based on phylogenetic analysis, it is possible to speculate the nature of energy metabolism of microorganisms present in a specific bacterial community [21]. We have found that most of the TSB sequences in different taxonomic divisions could be related to representatives with known metabolic traits. Organisms with such physiological properties could, therefore, be expected to reside in Tantloi hot spring sediments. Relatedness to *Thermus* and *Thermoanaerobacter* suggested that some organisms might have the capability to reduce Fe(III), Mn(IV), Co(III), U(VI), SO_4_^2-^ and Cr(VI) while the presence of *Thermodesulfovibrio*- and *Aquifex*-related bacteria indicated sulfate reduction. Interestingly, closely related bacteria *Thermus* SA-01 and *Thermus* sp. NMX2 A.1 are capable of reducing different metals including chromium [19]. Although the sediment samples collected from the non chromium(VI) contaminated source, cultures enriched in presence of Cr(VI) were found to reduce chromium both aerobically and anaerobically. Our previous study demonstrated similar finding with the isolate TSB-6 [20]. However, sequence analysis of rRNA gene library clones derived from the enrichment consortia revealed the abundance of family *Bacillaceae*. Further, 10 out of the 11 isolates obtained by purification on LB-agar medium containing 5 mM Cr(VI) were affiliated to the genus *Anoxybacillus*, the remaining one showed 97% similarity to *Bacillus*.

Thus, the native sediment samples of Tantloi hot streams, although predominated by *Thermus*, yielded on enrichment mostly *Anoxybacillus* isolates. Encouragingly, the enrichment consortia as well as all the isolates had a remarkable capacity to reduce Cr(VI) aerobically at high temperatures. The consortia could reduce up to an initial concentration of 20 mM Cr(VI) whereas a few of the isolates reduced an initial concentration of 5 mM Cr(VI) completely in 6 days.

Efficient microbial reduction of chromium has been earlier reported at high temperatures especially under anaerobic conditions. An anaerobic hyperthermophilic archaeum, *Pyrobaculum islandicum*, reduces 400 μM of Cr(VI) completely within 2 h [22]. Two strains of the genus *Thermus*, SA-01 and NMX2.A1, are known to reduce a number of metals including chromium at 65°C [19]. It is interesting to note that most strains of *Thermus* have been classified as obligate aerobes [23]. However, *Thermus* strains SA-01 and NMX2.A1 are facultative anaerobes and, in fact, were shown to reduce Cr(VI) and other metals under anaerobic conditions - 120 μM Cr(VI) is reduced completely in 20 h [19]. A thermophilic strain TOR 39 of *Thermanaerobacter* can anaerobically reduce chromium at 65°C, however, at initial Cr(VI) concentrations below 1 mM [24].

Chromium reducing consortia have been enriched earlier from a non-contaminated source under mesophilic conditions by Bader *et al.*[25]. We have, in addition to the enrichment of chromium-reducing consortia from a non-contaminated source at a high temperature, carried out a molecular analysis of the native and enriched microorganisms and identified the pure isolates. It has been speculated that microorganisms present in such environments may reduce chromium by enzyme(s) with a completely unrelated primary physiological role [25].

Localization of the chromate reduction activity in the pure isolates indicated certain similarities of these with a number of *Pseudomonas* strains [26-28] which possess a soluble reductase. There are other strains, however, [28,29] where the reduction activity is membrane associated. A *Bacillus* strain has also been reported to possess a soluble Cr(VI)-reducing enzyme that is stimulated by copper [30]. It may be noted that consistent with the significant whole-cell reduction activity of TSB-1, the crude extract as well as the soluble fraction of the isolate also possessed a very high specific activity.

The results from the present study showing thermophilic bacteria efficiently reduce high concentrations of chromium under aerobic conditions have important future prospects. First, anaerobic conditions would be difficult to achieve in soils [25]. Second, it has been speculated that under anaerobic soil conditions with high electron pressure microbes may play little direct role in the chemical reduction of Cr(VI) by Fe(II) or sulfides, but aerobic conditions with low reducing agent concentrations would require microorganisms to play an important role [31]. Besides, aerobic microorganisms usually being more amenable to mutation studies and genetic manipulations on these strains may help elucidate the mechanisms of chromium reduction at high temperatures. Our isolates also have considerable potential for bioremediation at high chromium concentrations which, in cases of several other Cr(VI)-reducing bacteria, have shown pronounced growth inhibition.

## Conclusions

In this study, 16S rRNA gene clone library based analysis was performed to understand the bacterial diversity present in a hot spring in Tantloi, India. No, such detailed phylogenetic analysis of the bacterial community is yet reported from this hot spring ecosystem. The analysis revealed that Tantoli hot spring sediment harbored a phylogenetically diverse bacterial population represented by at least ten taxonomic divisions. As approximately 80% of the sequences obtained in this study represented novel phylotypes that had less than 97% similarity with known sequences, many novel and useful extremophilic microbes are expected to be discovered from this hot spring in the near future. Further, The hot spring that we have explored in this study, though not a chromium-contaminated site, contains thermophilic *Bacillus* and *Anoxybacillus* strains with highly efficient aerobic Cr(VI)-reducing activity. Chromate reduction in some of the isolates obtained in this study is found to be mediated by a soluble cytoplasmic enzyme. These isolates should enable purification of reductases with high specific activity. Chromium bioremediation strategies at high temperature could also be designed utilizing the isolates obtained and/or enzymes isolated from them.

## Methods

### Sediment samples

Sediment samples were collected from a hot spring in Tantloi, situated in a region bordering West Bengal and Jharkhand states of India in sterile polypropylene tubes. The temperature and pH of the sediment at the site of collections were determined respectively by a digital pH meter and a temperature recorder. Portions of the samples were stored in 10% glycerol for enrichment and pure culture isolation. Samples were labeled properly and brought to the laboratory on ice. The total chromium content of soil samples was determined by Atomic Absorption Spectroscopy after acid digestion of the dried and powdered samples with hydrofluoric and perchloric acid (Modification of EPA method-3052) [32]. Cr(VI) concentrations were determined colorimetrically at 540 nm by reaction with diphenyl carbazide reagent after hot alkali digestion of samples as described in EPA method 3060A [33]. Free Cr(VI) in water and soil samples was measured by diphenyl carbazide reaction after extraction with deionized water [34].

### DNA extraction

DNA was extracted from the sediment samples according to the procedure described by Yamamoto *et al*. [35] with slight modifications. Briefly, ca. 5 g (wet weight) of the soil sample was suspended in 10 ml soil buffer (50 mM Tris.Cl, 200 mM NaCl, 5 mM EDTA, 0.05% Triton X-100 at pH 8.3 ). After centrifugation the pellet was washed with 10 ml soil buffer without Triton X-100 and resuspended in 5 ml of lysis buffer (10% Sucrose, 0.7 M NaCl, 40 mM Na_2_EDTA, 50 mM Tris.Cl, pH 8.3). Lysozyme was added to a final concentration of 1 mg ml^-1^. The suspension was subjected to 3 cycles of disruption by freezing at - 65°C and thawing at + 65°C. The samples were then treated with 50 μg ml ^-1^ proteinase K at 55°C for 1 h followed by 1% SDS at 55°C for 1.5 h with gentle mixing by inversion every 15–20 min. The slurry was further treated with 1% hexadecyltrimethyl ammonium bromide (CTAB) at 55°C for 30 min. The lysate was extracted once with equal volume of phenol-chloroform (1:1) and once with chloroform. Nucleic acids were recovered by ethanol precipitation. After removal of RNA by RNase digestion, DNA was passed twice through QIAGEN Genomic Tip 100/G to eliminate humic contaminants.

### Analysis of 16S rRNA genes

1 pg to 50 ng of the total community DNA was amplified in a 50 μl reaction mixture containing 5 μl of 10 × PCR buffer (MBI, Fermentas), 45 pmol of each primer, 0.2 mM of each of the four dNTPs and 2.5 U of Taq polymerase (MBI, Fermentas) using bacteria-specific forward primer 5′-AGA GTT TGA ACA TGG CTG-3′ (S-D-Bact-0027-a-S-18) and reverse primer 5′-CTA GCG ATT CCG ACT TCA-3′ ( S-D-Bact-1327-a-A-18) [36]. The numbers refer to the positions in the *Escherichia coli* 16S rRNA [37]. Reaction mixtures were incubated in a thermal cycler (GeneAmp 2400 PCR system, PE Applied Biosystems) for an initial denaturation at 94°C for 2 min followed by 40 cycles of 94°C for 1 min, 50°C for 1 min and 72°C for 2 min. Amplified DNAs were gel purified (QIAGEN Gel Extraction kit, QIAGEN, Germany). The purified DNAs were cloned directly by the TA cloning method (30) with a pGEM-T easy vector system II kit (Promega). The clones were sequenced using vector specific primers in an ABI Prism 3100 (16 capillary) sequencer (Applied Biosystems) according to manufacturer's instruction.

### Phylogenetic analyses

All sequences were edited manually and trimmed to remove ambiguous regions using BioEdit software [38]. Only sequences which read ≥ 500 bp were used in the analyses. Sequences were aligned among themselves by Clustal W multiple sequence alignment program to check their uniqueness. The CHECK_CHIMERA of RDP [39] and Bellerophon [40] programs were used to identify possible chimeric sequences which were then removed from further analysis. Sequences were examined for their approximate phylogenetic affiliations and orientation by employing BLAST program. Further, these sequences together with that of their closest relatives, as downloaded from databases, were aligned by Clustal W. Phylogenetic analyses were restricted to nucleotide positions that could be unambiguously aligned in all sequences and a neighbor-joining tree was constructed with MEGA 3.1 package [41]. The archeon *Methanobacteria* was used as the outgroup. Bootstrap analysis (1000 replicates) was performed with Kimura-2-parameter model to obtain confidence estimates for the phylogenetic tree.

### Cr-reducing enrichment consortia

Sediment samples were inoculated in Luria-Bertani (LB) broth (Difco) supplemented with different Cr(VI) concentrations ranging from 0.3 to 5 mM and incubated at 65°C. The source of Cr(VI) in this study was K_2_CrO_4_, prepared as 1 M stock solution, sterilized by filtration and added just prior to inoculation. There were three controls: (a) medium inoculated with autoclaved soil and (b) uninnoculated medium containing appropriate concentrations of Cr(VI) in order to rule out abiotic reduction, and (c) consortia without chromium to determine the inhibitory effect of Cr(VI) on bacterial growth, if any. Growth was monitored by A_600_ measurements [32] while Cr(VI) reduction (residual chromate measurement) was assayed by S-diphenyl carbazide method as described previously after removing the cells by centrifugation and diluting the supernatant as required. DNA from 2 day old Cr(VI)-reducing aerobic enrichment culture was purified as described previously for soil DNA except for the fact that washing was done twice with STE buffer only. Subsequently, 16S rRNA gene library was constructed from this DNA, clones were sequenced and phylogenetic analyses with 1025 bootstrap replicates were carried out. Trees were calculated by maximum parsimony, neighbor joining and minimum evolution algorithms in MEGA 3.1 package.

### Pure culture isolation

For pure culture isolation, Cr(VI) reducing enrichment culture was diluted, plated on LB-agar (3%) medium containing 5 mM of Cr(VI) in Hungate tubes and incubated for 2 days at 65°C [20]. Colonies were transferred thrice on similar medium and 11 isolates growing well on 5 mM Cr(VI) supplemented medium were subjected to phylogenetic analysis as described earlier and their Cr(VI)-reducing activity studied.

Cr(VI) reduction and corresponding growth rate at various Cr(VI) concentrations were studied by A_540_ and A_600_ measurements respectively. Isolates were also transferred on LB-agar medium containing 10 mM, 20 mM and 30 mM Cr(VI) to observe the tolerance of the isolates to high Cr(VI) concentrations.

### Cell fractionation and chromium reduction

Cells from overnight cultures were harvested by centrifugation at 4000 g for 10 min, washed and resuspended in 50 mM Tris–HCl buffer, pH 7.0. They were disrupted in an ice bath with an ultrasonic probe (Labsonic M, Sartorius, Göttingen, Germany). The sonicate was centrifuged at 13,000 g for 15 min at 4°C to remove cell debris and unbroken cells. A portion of this cell-free extract was centrifuged at 150,000 × g for 1 hour at 4°C. The supernatant thus produced was the soluble fraction while the pellet resuspended in 50 mM Tris–HCl buffer, pH 7.0 used as the membrane fraction [20]. Protein concentration was estimated by Lowry method using bovine serum albumin as standard.

Chromium reduction was studied by adding equivalent amounts of crude cell extract, soluble fraction and membrane fraction to the reaction mixtures containing 50 mM Tris–HCl, pH 7.0, 50 μM K_2_CrO_4_ and 0.2 mM NADH. The total reaction volume was 2 ml and tubes were incubated at 65°C. Aliquots were removed at regular time intervals and Cr(VI) remaining was measured spectrophotometrically by S-diphenyl carbazide method as mentioned earlier. Reduction was also studied at higher initial chromium concentrations of 100 μM, 200 μM and 500 μM.

### Nucleotide sequence accession numbers

The accession numbers of 16S rRNA gene sequences determined in the present study and deposited in GenBank are as follows: EF017721-EF017765 (native), EF017767-EF017784 (enrichment) and EF017785-EF017795 (pure isolates).

## Competing interests

The authors declare that they have no competing interests.

## Authors’ contributions

PJ carried out the experiments, molecular analyses and participated in the research design. HMR discussed the analyses, presentation of data and critically reviewed the manuscript. SP provided supervision, participated in the research design and drafting the manuscript. All authors read and approved the final manuscript.
